# Sensorineural Organs Dysfunction and Cognitive Decline: A Review Article

**DOI:** 10.14336/AD.2016.0515

**Published:** 2016-12-01

**Authors:** Supakanya Wongrakpanich, Aisawan Petchlorlian, Andrew Rosenzweig

**Affiliations:** ^1^Department of Medicine, Einstein Medical Center, Philadelphia, Pennsylvania, USA and Department of Medicine, Chulalongkorn University, Thailand; ^2^Division of Geriatric, Department of Medicine, Chulalongkorn University, Thailand; ^3^Division of Geriatric, Department of Medicine, Einstein Medical Center, Philadelphia, Pennsylvania, USA

**Keywords:** cognitive decline, olfaction loss, vision loss, hearing loss, aging

## Abstract

Vision, hearing, olfaction, and cognitive function are essential components of healthy and successful aging. Multiple studies demonstrate relationship between these conditions with cognitive function. The present article focuses on hearing loss, visual impairment, olfactory loss, and dual sensory impairments in relation to cognitive declination and neurodegenerative disorders. Sensorineural organ impairment is a predictive factor for mild cognitive impairment and neurodegenerative disorders in the elderly. We recommend early detection of sensorineural dysfunction by history, physical examination, and screening tests. Assisted device and early cognitive rehabilitation may be beneficial. Future research is warranted in order to explore advanced treatment options and method to slow progression for cognitive declination and sensorineural organ impairment.

Ears, eyes, and noses are sensorineural organs. Their functions play important role for quality aging. Presbyopia, presbycusis, as well as presbyosmia, are known to be age-related conditions. These conditions have been studied in relation to cognitive impairment and neurodegenerative disorder.

Cognitive functioning is essential for independent living and successful aging[1]. Based on a systematic review of prevalence studies for dementia, it has been estimated that dementia exists in 1.5% of adults age 65 to 69 and increased to 24.8% in adults age 85 and older[2]. Alzheimer’s disease, Lewy body dementia and vascular dementia are three most common causes of dementia[3, 4].

To our knowledge, this is the first review that examines each sensorineural organ dysfunction; including, vision loss, hearing loss, and olfactory loss, and their correlation with cognitive impairment. In this review, we also focus on pathophysiology of the relationship, current available treatment options, and future research trends.

## Hearing loss

Hearing impairment is the most common sensory deficit with prevalence reported to be as high as 64.1% in the population age above 65 years old[5, 6]. Since Uhlmann et al. promoted a hypothesis that a relationship exists between hearing impairment and Alzheimer’s type dementia in the elderly[7], multiple sources have demonstrated a clear association between these two conditions[1, 6, 8-11]. Both peripheral type[8] and central type[12] hearing loss have been linked to dementia. In 2013, Linn et al. did a prospective observational study in 1,984 older adults. They did audiometric testing at year 5 and cognitive testing at year 5, 8, 10, 11. They found that participants with hearing loss had an increased risk for cognitive impairment[13]. The causative link between these two conditions is still unclear. Whether the hearing loss is a cause and/or aggravating factor of cognitive decline or vice versa is not yet known[5].

Several explanations about the association between hearing impairment and cognitive decline have been offered. First, hearing impairment is not only a deficit in speech reception, but also includes a wide range of abilities mediated by hearing including spatial perception, divided attention in noisy environment, perception of environmental sounds, selecting relevant information, and comprehending the selected information in the temporal lobe[14]. All of these processes require cognitive processing. Second, cognitive testing performance may be skewed by the poor verbal communication that associated with hearing loss[13]. Third, there may be a shared neuropathophysiologic pathway between cognitive decline and hearing loss. Fourth, when limited cognitive abilities are present, a greater proportion of cognitive resources are required for auditory perceptual processing. This causes decreased resources for other cognitive domains, such as short-term memory.[11] Fifth, prolonged decreased adequate auditory neuronal input from hearing impairment can lead to neuronal atrophy and cognitive deterioration. This hypothesis is called “sensory deprivation”[1]. Lastly, a number of studies have confirmed the hypothesis that hearing impairment contributes to reduced quality of life, social isolation, and/or depression in the elderly, especially in those with suboptimal social support, thus resulting in accelerated cognitive decline[1, 11, 13, 15].

Simple otoscopic examination and cerumen disempaction can significantly improve hearing function in up to 10% of the elderly[16]. Auditory rehabilitation by hearing aids and cochlear implants is recommended in all elderly with hearing impairment. In the United States, hearing aids are used in 40% of adults with moderate hearing loss and 3.4% of those with mild hearing loss[17].

It is still controversial whether hearing aid slows the progression or reverses the clinical course of cognitive impairment[6]. In 1990, Mulrow et al. conducted a randomized trial about hearing impairment and quality-of-life changes. They found that the improvement of cognitive function was seen in subjects with hearing aids[18]. Later, Choi et al. found that short term memory and learning ability of individuals with hearing impairment improved after using hearing aids[19]. Acar et al. found significant improvements in psychosocial and cognitive conditions in older adults with hearing aids[20]. In 2003, Allen et al. studied the effect of improving hearing in dementia and found that, despite positive acceptance rates of hearing aid usage, it did not improve cognitive function or reduce behavioral or psychiatric symptoms [16]. In 2014, Wong et al. conducted a prospective study in 34 older adults and found that, despite appropriately fitted hearing aids, they had significant benefits only for improve sensitivity to sound and speech understanding. However, cognitive decline was still significant and not fully compensated by hearing aids[21].

The future research includes further understanding on underlying pathophysiology of an association between hearing loss and cognitive decline, as well as effective method of auditory rehabilitation.

## Visual Impairment

Visual impairment occurs in 9% to 18% of the elderly[22]. In the UK, approximately 5% of ophtalmologic outpatients age 60 and above are expected to have dementia[23]. A relationship between age-related visual disorders and cognitive decline has been recognized in the literature. Apart from presbyopia, which is considered to be a universal age-related vision change, cataract, glaucoma, and macular degeneration are very common visual problems in the elderly[24]. A study by Elliot et al. reported that the prevalence of visual disturbances in assisted living facilities is as high as 90% in adults age 60 and above[25]. In 2007, a systematic review from nine cohort studies by Tas et al. demonstrated that vision impairment as well as cognitive impairment are prognostic factors for disability in older people[26]. In 2009, Rogers et al. demonstrated that poor vision is associated with dementia development. In addition, they found that poor vision without a previous eye procedure put the elderly at 5 times risk of Alzheimer’s disease development[26]. Interestingly, visual disturbance can also be the presenting manifestation of Alzheimer’s disease[26]/.

### Age-related macular degeneration (ARMD)

Macular degeneration occurs approximately 39% to 47% in adult age above 65 years old[27]. From population-based cross-sectional study of approximately 9,000 older individuals by Wong et al. in 2002, people with severe cognitive impairment were more likely to have age-related macular degeneration[28]. Similar results were found in a population-based study of 2,000 individuals by Baker et al.; people with cognitive function decline were more likely to have early ARMD (odds ratio 1.38; 95% confidence interval, 1.03-1.85)[29]. In addition, Pham et al. found an cross-sectional association between late ARMD and cognitive impairment in older Australians.[30] However, the association was weaker when removing vision-related items from the mini mental status examination (MMSE) instrument.

### Glaucoma

Glaucoma occurs in 12% to 13% of individuals age greater than 65 in the UK[27]. Alzheimer’s disease and glaucoma share some similarities. They are slow progressive chronic neurodegenerative disorders with a strong age-related incidence[31]. Wostyn et al. proposed a causal relationship between glaucoma and Alzheimer’s disease. They hypothesized decreased cerebrospinal fluid pressure causing abnormal high trans-lamina cribrosa pressure difference may put Alzheimer’s disease patient at a greater risk of developing glaucoma[31].

### Cataract

Cataract has a prevalence of 11% to 25% in the elderly[23, 27]. Also, increased number of severe cataracts was reported in elderly populations[23]. Despite knowing that cataract has some associations with cognitive impairment, an interesting future research question is whether cataract surgery improves cognitive performance? Despite a strong association between vision loss and cognitive declination, visual-enhancing interventions including cataract surgery or refractive error correction have not shown a clear benefit in improving cognition function. Results from recent studies are contradictory. Gray et al [32], Tamura et al[33], and Ishii et al[34] did prospective observational studies and found that there was an improvement in cognitive score after cataract surgery. In contrast, Hall et al[35], Elliot et al[36], and Anstey et al[37] found that there was no significant difference in cognitive score before and after cataract surgery.

There are four possible hypotheses for cognitive declination related to visual impairment. First, these two conditions are both neurodegenerative disorders that are associated with aging, and may share common pathogenic pathways.[28] Second, Jefferis et al[23] hypothesized that visually impaired patients were unable to see and interpret the cognitive testing material causing lower score. Also, Killen et al. did a case-control study in 15,033 elderly patients and found that patients with visual impairments performed poorer than a normal cohort in the vision-dependent items in cognitive screening tests. They recommended that patients with visual impairments would benefit from cognitive tests, which do not rely on vision[38]. Third, cognitive decline and some forms of visual impairment share the same risk factors, diabetes, smoking, and obesity, which are considered risk factors for age-related cataract and dementia[39, 40]. Fourth, hyperphosphorylated tau and amyloid-β protein, which are considered as neurotoxic substance, have been found in both senile dementia and glaucoma, suggesting of common pathophysiologic mechanisms[41].

### Future trends

Whitson et al. did a pilot study in 12 patients and demonstrated that low visual rehabilitation has been shown to improve cognitive measures in mild cognitive deficit patients with low vision[42]. For Glaucoma, targeting amyloid-β protein formation and aggregation pathway, which response for glaucoma and dementia, can reduce glaucomatous retinal ganglion cells apoptosis *in vivo*[41, 43]. In addition, it has been found that immunotherapy targeting amyloid-β protein can improve cognitive function in some patients[41, 44].

### Olfactory loss

With a prevalence of more than 50% in individuals aged between 65 and 80 years, and up to 80% in those age above 80 years, olfaction dysfunction is considered a very common problem in the elderly[45]. Like presbyopia and presbycusis, presbyosmia or age-related olfactory loss can lessen the quality of life of the elderly. Also, it can be a predictor of overall mortality in the elderly[46, 47]. There are multiple factors that responsible for presbyosmia. These include olfactory epithelium damage from environmental insults, age-related nasal epithelium atrophy, decrease in nasal mucosal metabolizing enzymes, sensory loss of olfactory neuronal receptor cells, age-related decrease in number of odorant-selective receptor cells and first-order neurons, reduction in foramina in the cribriform plate, and changes in olfactory neurotransmitter system[45].

Noteworthy is the fact that olfactory identification deficit is one of the earliest markers that strongly predict the development of mild cognitive impairment and neurodegenerative diseases including Alzheimer’s disease, Lewy body dementia and Parkinson’s disease. This has been confirmed by multiple studies[45, 48-55]. Later in 2014, Sanke et al. demonstrated same persistent findings in type 2 diabetes mellitus elderly with cognitive impairments[56]. In addition, the severity of olfactory seems to correlates substantially with the severity of dementia[52]. As a result of the relationship between olfactory identification deficit and cognitive impairment, smell identification test is considered to be the feasible test to detect mild cognitive impairment and Alzheimer’s disease in community dwelling elders[57-59].

Possible pathophysiologic links between olfactory loss and cognitive dysfunction can be elucidated by four hypotheses: varies from genetic aspect, genomic expression, and clinical point of view. First, ApoE-ε4 allele, which is believed to play critical role for Alzheimer’s disease development, was found to be associated with odor identification impairment in the elderly[60, 61]. In addition, olfactory dysfunction in the presence of ApoE genotype is a risk factor for cognitive decline[62]. Second, Tau protein and amyloid-β protein, which are key pathophysiological substances for Alzheimer’s process, have also been found in olfactory pathway as well. In 2006, Attems et al. did a study of autopsy cases with Alzheimer’s disease. They did neuropathological assessment with immunohistochemical study and found tau protein deposits in the olfactory bulb and nerve[63]. Later, in 2012, Nelson et al. found that Alzheimer’s disease also has amyloid-β protein deposits at medial temporal lobes, which is also olfactory pathway[55, 64]. Third, hippocampal area is considered an anatomical brain location that responsible for memory as well as odor memory. There are evidences of reduction in the size of hippocampus from neuroimaging related to odor identification in Alzheimer’s patient.[65, 66] Fourth, olfactory loss can be directly or indirectly affect quality of life such as enjoyment of food and everyday safety, and eventually can cause depression, which can lead to cognitive declination[45, 67].

### Future trend

There is limited data about the feasible treatments for olfactory loss, as well as, improvement of cognitive function after improved olfactory function. Sinus disease seems to be the only condition of olfactory loss, which responds well to treatment. Environmental and lifestyle modification may also help with the patient with anosmia, including, gas detector installation, and labeling the date for leftover food[68]. However, the existing literature revealed some evidence that the olfactory system may be a feasible treatment option of mild cognitive impairment and neurodegenerative disorders. Chen et al. conducted an animal study by given recombinant human nerve growth factor to the brain via the olfactory neural pathway. They found a satisfactory amount of growth factor in the brain by this noninvasive nasal drug delivery method[69]. Salvinelli et al. also conducted a trial with novel technique for drug delivery called ‘high-pressure administration of sterile physiological saline isotonic solution’ that found to trigger endogenous enhancement for nerve growth factor[70]. Later, Craft et al. conducted a randomized-controlled trial with intranasal insulin administration and found that intranasal insulin improved delayed memory and preserved caregiver-rated functional ability in mild cognitive impairment patient[71]. A potential beneficial cognitive effect of intranasal insulin without any major side effects was confirmed by a systematic review by Shemesh et al. in 2012[72]. Also, a combination of high-dose vitamin D with intranasal insulin was introduced by stein et al[73], however, the combination regimen did not show any significant cognition benefit over low dose vitamin D in Alzheimer’s disease.

## Dual Sensory Impairments

Frequently, geriatric patients have sensorineural dysfunction across multiple organs. Dual sensory impairments, which were defined by a combination of vision and hearing loss, has the prevalence of approximately 5% to 21%[22, 74, 75]. There is an established relationship between dual sensory impairments and functional disability among elderly individuals. In 2001, Wallhagen et al. did a 1-year prospective cohort study in community indwelling older adults who had dual sensory impairments and found that hearing and vision loss had strong independent impacts on functioning abilities, including ADL and social functioning[76]. In addition, dual sensory impairments have a greater impact on function than single sensory impairment[77]. Also, dual sensory impairment was associated with greater instrumental activities of daily living defecits than in single sensory loss[22]. Because of the difficulties for activities of daily living, dual sensory loss patients can develop depressive symptoms, which may related to worsening cognitive function[78].

There is a paucity of evidence for cognitive decline related to dual sensory impairments when compared with single sensory impairment. In 2005, results from the Maastricht Aging Study demonstrated an association between changes in auditory and visual acuity, and changes in cognitive functioning[1]. Humes et al[79] conducted a study in 2012 about sensory processing, including tactile, vision, and hearing. They found that cognitive declination may resulted from age related changes in global sensory processing.

### Future trend

A screening tool for dual sensory loss do exist and easy to use[80]. However, to date, there is no evidence about visual changing and hearing aids on cognitive improvement. Future research should also focus on an effective combined rehabilitation program.
